# Systemic and intestinal porcine epidemic diarrhea virus-specific antibody response and distribution of antibody-secreting cells in experimentally infected conventional pigs

**DOI:** 10.1186/s13567-020-00880-z

**Published:** 2021-01-04

**Authors:** Yuto Suda, Ayako Miyazaki, Kohtaro Miyazawa, Tomoyuki Shibahara, Seiichi Ohashi

**Affiliations:** 1 Kyushu Research Station, National Institute of Animal Health (NIAH), National Agriculture and Food Research Organization (NARO), 2702 Chuzan, Kagoshima, Kagoshima 891-0105 Japan; 2 Division of Viral Disease and Epidemiology, NIAH, NARO, 3-1-5 Kannondai, Tsukuba, Ibaraki 305-0856 Japan; 3 Division of Pathology and Pathophysiology, NIAH, NARO, 3-1-5 Kannondai, Tsukuba, Ibaraki 305-0856 Japan; 4 Department of Veterinary Science, Graduate School of Life and Environmental Sciences, Osaka Prefecture University, 1-58 Rinku-oraikita, Izumisano, Osaka 598-8531 Japan

**Keywords:** porcine epidemic diarrhea virus, mucosal immune responses, antibody-secreting cells

## Abstract

Porcine epidemic diarrhea (PED) is a coronavirus disease characterized by the rapid spread of severe diarrhea among pigs. PED virus (PEDV) infects and replicates mainly in the epithelial cells of the duodenum, jejunum, ileum and colon. Serum or mucosal IgA antibody levels have been used to predict both vaccine efficacy and the level of protective immunity to enteric infectious diseases in individuals or herds. Details of the B-cell immune response upon PEDV infection, such as the systemic and mucosal PEDV IgA antibody response, the distribution of IgA antibody-secreting cells (ASCs), and their role in virus clearance are not yet clear. In this experimental infection study, we observed similar fluctuations in PEDV IgA antibody levels in serum and intestinal contents of the upper and lower jejunum and ileum, but not fecal samples, over the 4-week experimental course. ASCs that actively secrete PEDV IgA antibody without in vitro stimulation were distributed mainly in the upper jejunum, whereas memory B cells that showed enhanced PEDV IgA antibody production upon in vitro stimulation were observed in mesenteric lymph nodes and the ileum. Our findings will contribute to the development of effective vaccines and diagnostic methods for PEDV.

## Introduction

Porcine epidemic diarrhea (PED) is an emerging and re-emerging swine disease caused by PED virus (PEDV), a member of the family *Coronaviridae*, genus *Alphacoronavirus*. PED is characterized by the rapid spread of acute and severe diarrhea within pig herds. Mortality reaches nearly 100% in suckling piglets at susceptible seronegative farms [1–3]. With age, however, the severity of clinical signs becomes milder and the mortality rate decreases, although older pigs are still susceptible to viral infection. After its appearance in 1974, PED caused losses mainly in European and east Asian countries up until the 1980s. From the 1990s to 2000s, however, the virus was problematic in east Asian countries such as China and Korea, but not in Europe. From 2013, new highly pathogenic PEDV strains that emerged in 2010 in China [4] have spread to North and South American countries where PED had not reported before [5–8] and re-emerged in Asian countries [9–12]. A single outbreak of the pathogenic strain has also reported in Ukraine [13]. Approximately 8 million pigs died in the United States during the 2013 to 2014 epidemic [14]. PED is now considered an important emerging and re-emerging diseases that causes severe economic loss to the swine industry [15].

PEDV is transmitted by the fecal–oral routes and replicates mainly in the epithelium of the small intestine (especially in the jejunum and ileum) and colon [16]. Viral replication causes vacuolization and destruction of cells, leading to villous atrophy and watery diarrhea [17, 18]. Although the PEDV genome is often detected in serum from infected suckling and weaned pigs [16, 19–21], active viral replication in cells other than intestinal epithelial cells has yet to be reported in pigs [16, 18].

For enteric viruses that replicate mainly in intestinal epithelial cells, locally produced virus-specific IgA antibodies are the most important function for the primary adaptive defense at mucosal surfaces [22, 23]. While serum neutralizing and IgG antibody responses are rarely correlated with protection against infection and onsets of most enteric virus infections [22, 24–26], serum or mucosal IgA antibody levels have been used to predict both vaccine efficacy and the immune status of individuals or herds against enteric infectious diseases such as rotavirus infection [25]. However, despite the wide use of serum PEDV IgA antibody testing to assess infection and vaccine immunity against PEDV [27, 28], few studies have investigated the relationship between serum and intestinal PEDV antibody responses.

The mucosal immune system is unique in that it includes both induction sites such as Peyer's patches and mesenteric lymph nodes (MLNs), where naïve B cells are activated and differentiate into antibody-secreting cells (ASCs) and memory B cells, and effector sites such as the lamina propria of the small intestine, to which ASCs migrate and secrete antibodies to induce immune-exclusion at the mucosal surface [22, 29–31]. The elucidation of not only antibody levels in serum and body fluids but also the distribution of ASCs is therefore fundamental to understanding the intestinal immune system for vaccine development and improvement. The distribution of ASCs in systemic and mucosa-associated lymphoid tissues of PEDV-infected pigs has been evaluated [32]. However, the relationship between PEDV antibody levels in serum and those in intestinal contents remains unclear. Further, details of the distribution of PEDV-specific ASCs and their antibody production in the small intestine are also unclear.

Here, we analyzed the kinetics of antibody responses to PEDV in serum and intestinal contents in an experimental PEDV infection study of 4-week-old pigs. We further investigated the distribution of PEDV-specific ASCs in the upper and lower jejunum and ileum as well as in blood and spleen to understand the local immune responses in PEDV-infected pigs.

## Materials and methods

### Cells and viruses

Vero cells were cultured in Eagle's minimum essential medium (EMEM, Nissui Pharmaceutical, Tokyo, Japan) supplemented with 5% fetal bovine serum (FBS, Thermo Fisher Scientific, Waltham, MA, USA) at 37 ℃ with 5% CO_2_. The PEDV strain OKN-1/JPN/2013, a Japanese strain in Group II that was isolated from a homogenate obtained from a diarrheal piglet in 2013 [33], was propagated in Vero cells in EMEM supplemented with 10 μg/mL trypsin 1:250 (Gibco, Grand Island, NY, USA) and used as a virus inoculum. Pigs were inoculated with a dose of 3 × 10^5^ TCID_50_/2 mL/pig at the 9^th^ cell passage.

### Study design

The animal experiment was approved and performed according to the regulations and guidelines of the Animal Ethics Committee of National Institute of Animal Health (NIAH), National Agriculture and Food Research Organization (NARO) ([18-078] and [18-088]). A total of 24 PEDV seronegative 4-week-old pigs were used; 16 were orally inoculated with PEDV OKN-1/JPN/2013 and the other eight were used as negative controls. Fecal samples were collected directly from the rectum of each pig every day from 0 to 5 days post-inoculation (dpi) and twice a week from 7 to 28 dpi for quantification of viral RNA by reverse transcriptase polymerase chain reaction (qRT-PCR) and antibody detection.

Virus-inoculated pigs were euthanized at 2, 7, 14 and 28 dpi (n = 4 each dpi). The small intestine (upper jejunum and ileum), spleen, MLNs, and blood were collected for the isolation of mononuclear cells (MNCs). Serum and small intestinal contents (SICs) of the upper and lower jejunum and ileum were collected for antibody detection at necropsy. Among the eight control pigs, four were sacrificed for the isolation and analysis of MNCs. SICs were only available from six of the eight pigs in the control group because the small intestine was empty in the other two control pigs. After making a 20% suspension in phosphate-buffered saline (PBS) and centrifuging at 2000 × *g* for 15 min, the supernatants were collected and stored at − 80 ℃ until RNA extraction (fecal samples) and antibody detection (fecal and SIC samples).

### qRT-PCR for PEDV nucleocapsid gene

RNA was extracted from 250 µL of the 20% fecal suspension using ISOGEN-LS (Nippon Gene, Tokyo, Japan) according to the manufacturer's instructions. RNA was reverse transcribed and amplified using a One Step TB Green™ PrimeScript™ PLUS RT-PCR Kit (Perfect Real Time) (Takara Bio, Shiga, Japan) and an ABI 7500 Fast Real-Time PCR System (Thermo Fisher Scientific) according to the manufacturer's protocol. The primers used for qRT-PCR were as follows; forward: 5′-GAATTCCCAAGGGCGAAAAT-3′ and reverse: 5′-TTTTCGACAAATTCCGCATCT-3′ [34]. Viral RNA standards with known titers were used for quantification. The detection limit was determined to be 6000 genome copies/g.

### Indirect ELISA for PEDV-specific IgG and IgA antibody detection

The lysate of OKN-1/JPN/2013 or mock-inoculated Vero cells was prepared as a virus or control antigen for indirect enzyme-linked immunosorbent assay (ELISA), respectively. Vero cells (4 × 10^6^ cells) were inoculated with PEDV at an MOI of 1. After 24 h post-inoculation, the cells were lysed in 1 mL of 1% Triton X-100 (Nacalai Tesque, Kyoto, Japan) in PBS on ice for 30 min. Supernatants of the cell lysate were collected after centrifugation at 2000 × *g* for 10 min to remove cell debris and used as the virus ELISA antigen. The control antigen was prepared in the same manner using mock-inoculated cells. The microwell plates (Maxisorp, Thermo Fisher Scientific) were coated using the virus and control antigens, which were diluted to 1:2000 with coating buffer (15 mM Na_2_CO_3_ and 35 mM NaHCO_3_), overnight at 4 ℃. Lysates were not treated to remove Triton X-100 prior to use for coating plates. Virus or control antigen-coated plates were treated with blocking solution containing 5% skim milk (Morinaga, Tokyo, Japan) in PBS containing 0.1% Tween 20 (PBS-T) for 1 h at 37 ℃. Serum samples, 20% fecal suspension, and 20% SIC suspension were diluted 1:100, 1:2 and 1:16, respectively with PBS-T containing 5% skim milk and added to pairs of wells containing virus or control antigen. The plates were incubated for 1 h at 37 ℃ for serum, or overnight at 4 ℃ for fecal samples and SICs.

After a washing step with PBS-T, horseradish peroxidase-conjugated secondary antibodies against porcine IgG or IgA (Bio-Rad, Hercules, CA, USA) were added to each well, and the plates were incubated for 1 h at 37 ℃. The reaction was visualized by adding ABTS (2,2′-azino-bis (3-ethylbenzothiazoline-6-sulfonic acid)) substrate solution (Roche, Basel, Switzerland) to each well after a washing step. Optical density (OD) values were measured using an Infinite F50R absorbance microplate reader (TECAN, Männedorf, Zürich, Switzerland). Antibody levels are expressed as sample-to-positive (S/P) ratios: S/P ratio = ((OD value of sample in viral antigen well − OD value of sample in control antigen well)/(OD value of positive control sera in viral antigen well − OD value of positive control sera in control antigen well)). Positive control sera for detecting specific IgG and IgA antibodies were obtained from a sow that had been experimentally inoculated with PEDV OKN-1/JPN/2013. Cut-off values were determined to be the mean + three standard deviations of S/P ratios of serum and fecal samples collected from virus-inoculated pigs at 0 dpi or those collected from control pigs.

### Isolation of MNCs

MNCs were isolated from the blood, spleen, MLNs, and lamina propria of the small intestine (upper jejunum and ileum) as described previously [32, 35, 36] with some modification. Blood was aseptically collected into tubes containing EDTA 2Na (Terumo, Tokyo, Japan). Peripheral blood mononuclear cells (PBMCs) were isolated from 4 mL of blood by density gradient centrifugation using 60% Percoll (GE Healthcare, Chicago, IL, USA). PBMCs collected from the interface were washed twice with PBS and resuspended in RPMI-1640 (Nissui Pharmaceutical) containing 10% FBS and antibiotics: 100 μg/mL kanamycin (Wako Pure Chemical Corporation, Osaka, Japan) and 50 μg/mL gentamycin (Wako).

Pig spleens and MLNs were collected and placed in ice-cold wash buffer (RPMI-1640 containing antibiotics and HEPES (Wako); 10 mM). Spleens and MLNs were dissociated by pressing the tissues through a stainless-steel mesh. The cell suspensions were washed and centrifuged at 800 × *g* for 20 min at 4 ℃. After removing cell debris by gradient centrifugation in 33% Percoll at 1800 × *g* for 20 min at 4 ℃, MNCs were collected. MNCs were further enriched by additional collection at the interface following gradient centrifugation in 43% and 70% Percoll at 1800 × *g* for 20 min at 4 ℃. After a washing step with PBS, the MNCs were resuspended in RPMI-1640 containing 10% FBS and antibiotics, and the cell concentration was adjusted to 1 × 10^7^ cells/mL.

Small intestinal fragments (upper jejunum and ileum) of approximately 20 cm were collected aseptically and placed in ice-cold wash buffer. Peyer's patches were not removed for isolation of MNCs. The intestinal fragments were transferred to 40 mL of Hank's balanced salt solution without calcium and magnesium (Wako) but containing 40 mM HEPES, 5 mM EDTA (Wako), 1 mM DTT (Wako), 0.28% NaHCO_3_ (Wako) and antibiotics, and shaken at 190 rpm for 25 min at 37 ℃. The fragments were then washed and cut into small pieces. The pieces were suspended in 40 mL of RPMI-1640 containing antibiotics, 20 mM HEPES, 5 mM EDTA, 1 mM DTT, and 0.11% of type II collagenase (Thermo Fisher Scientific) and shaken at 190 rpm for 25 min at 37 ℃. Supernatants containing MNCs were collected, and the remaining tissues were pressed through a stainless-steel mesh for collection of additional MNCs. After removing cell debris by gradient centrifugation in 33% Percoll at 1800 × *g* for 20 min at 4 ℃, the MNCs were collected. MNCs were further enriched by collection from the interface following gradient centrifugation in 43% and 70% Percoll at 1800 × *g* for 20 min at 4 ℃. After a washing step with PBS, the MNCs were resuspended in RPMI-1640 containing 10% FBS and antibiotics, and the cell concentration was adjusted to 1 × 10^7^ cells/mL.

### Culture and in vitro stimulation of MNCs

MNCs isolated from blood and each tissue were suspended at 5 × 10^6^ cells/500 µL/well and cultured in RPMI-1640 containing 10% FBS and antibiotics with or without 1 μg/mL R848 (resiquimod) (AdipoGen Life Sciences, San Diego, CA, USA), which induces polyclonal activation of memory B cells [37]. Cells were added to each well of 24-well culture plates and incubated at 37 ℃ for 4 days in a 5% CO_2_ incubator. Cell culture supernatants were collected and analyzed for specific IgA and IgG antibodies by indirect PEDV ELISA.

### Statistical analyses

One-tailed Student's t-test, Dunnett's test, or Tukey's test were used to compare the differences in specific IgA or IgG antibody S/P ratios among the different time points or different organs. p-values of p < 0.10, p < 0.05, and p < 0.01 were considered to be suggestive, significant, and highly significant, respectively, for all comparisons. Statistical analyses were performed using Microsoft Excel and R. Correlation coefficients (r) between viral RNA copies (log copies/g) and antibody S/P ratios were calculated using Microsoft Excel. Viral RNA copy data from 0 dpi were omitted as they were not related to a decrease in viral shedding.

## Results

### Clinical signs and fecal viral shedding

**Figure 1:**
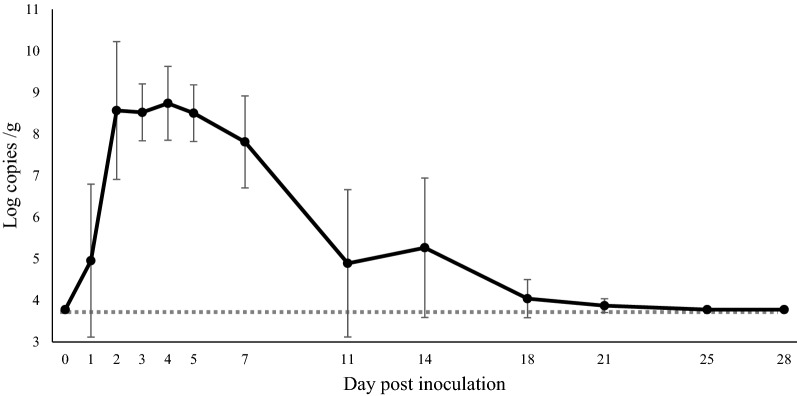
**Fecal shedding of PEDV RNA in inoculated pigs determined by qRT-PCR.** PEDV-seronegative 4-week-old pigs were orally inoculated with PEDV strain OKN-1/JPN/2013. Fecal samples were serially collected, and PEDV RNA was detected and quantified by qRT-PCR.

All 16 virus-inoculated pigs developed clinical signs such as decreased or complete loss of appetite, vomiting, and/or soft-to-watery feces from 1 to 7 dpi. At the same time that clinical signs were observed, fecal shedding of PEDV RNA peaked at around 2 to 5 dpi, and all inoculated pigs shed the virus (Figure 1). All 16 inoculated pigs recovered from the clinical signs, and the amount of detectable fecal PEDV RNA started to decrease after 7 dpi. The percentage of pigs that shed PEDV in feces also dropped off to 50% (4/8) at 11 dpi, 75% (6/8) at 14 dpi, and 25% (1/4) at 18 dpi and 21 dpi. Viral RNA could not be detected in feces after 25 dpi (Figure 1).

### S/P ratios of serum IgG and IgA and fecal IgA antibody against PEDV

**Figure 2:**
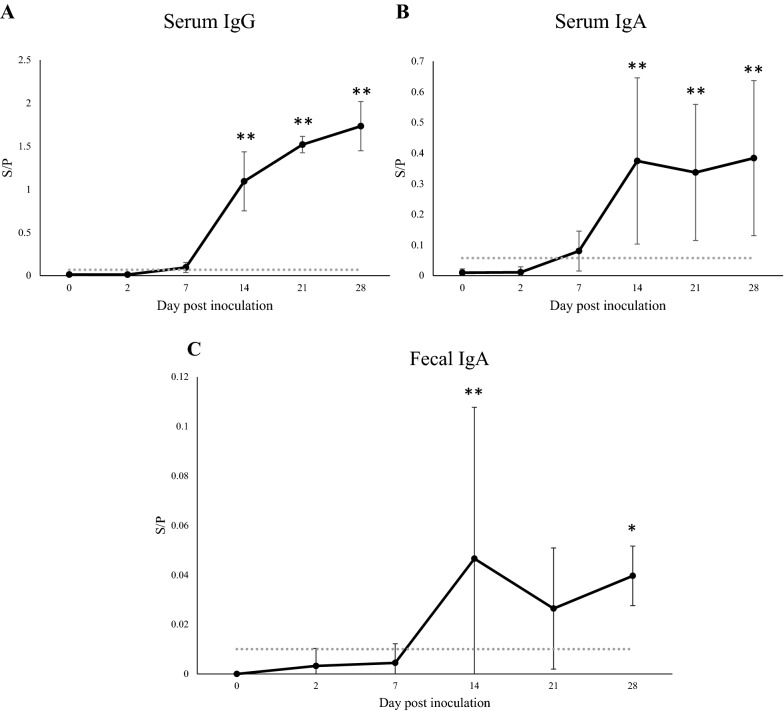
**S/P ratios of PEDV IgG and IgA antibodies in serum and fecal samples serially collected from pigs inoculated with PEDV.** Antibodies to PEDV in collected serum samples were detected by IgG (**A**) and IgA (**B**) ELISAs. Antibodies to PEDV in collected fecal samples were detected by IgA ELISA (**C**). *p < 0.05 and **p < 0.01 (significant and highly significant, respectively) determined using Dunnett's test. Cut-off values were the mean + three standard deviations of sample values at 0 dpi in infected pigs and negative controls. Solid line: PEDV-inoculated group. Dotted line*:* cut-off value.

Along with the diminished clinical signs and drop in fecal viral RNA shedding (decrease in both the number of copies of the viral RNA and the percentage of positive pigs), PEDV IgG antibody was detected in the serum of 66.7% (8/12) of inoculated pigs at 7 dpi. The S/P ratio of PEDV IgG antibody was significantly increased at 14 dpi compared to that at 0 dpi, and peaked at 28 dpi (Figure 2A). The correlation coefficient between the S/P ratio of serum PEDV IgG antibody and fecal virus shedding load was r = − 0.993 (p < 0.001). The PEDV IgA antibody S/P ratio showed a similar trend to that of the PEDV IgG antibody S/P ratio: PEDV IgA antibody was detected in the serum of 58.3% (7/12) of inoculated pigs at 7 dpi, and its S/P ratio peaked at 14 dpi and remained at the same level until the end of the study (28 dpi) (Figure 2B). The correlation coefficient between the S/P ratio of serum IgA and fecal viral load was r = − 0.952 (p = 0.012).

Although PEDV antibodies were detected in serum from all inoculated pigs at high S/P ratios after 14 dpi, they were only detected in the feces of 37.5% (3/8), 50% (2/4), and 100% (4/4) of inoculated pigs at 14, 21, and 28 dpi, respectively. The fecal S/P ratios of PEDV IgA antibodies were lower than those in serum (Figure 2B, C). In contrast, the S/P ratios of PEDV IgG and IgA antibodies from control pigs were below the cut-off value in both the serum and fecal samples throughout the study.

### Comparison of S/P ratios of PEDV IgA antibody in the SICs of the upper and lower jejunum, and ileum

**Figure 3:**
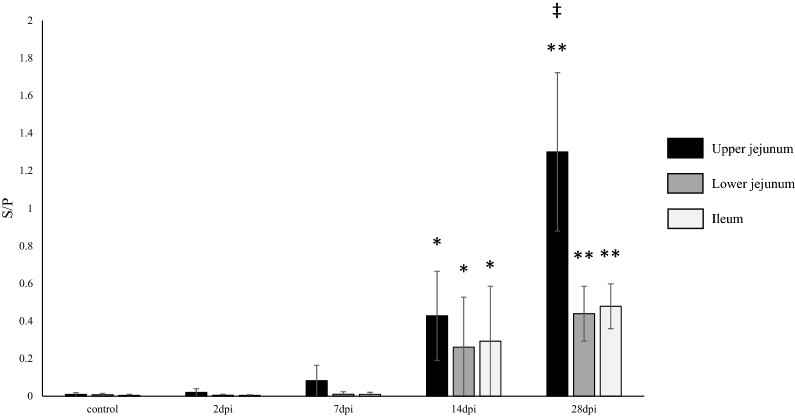
**S/P ratios of PEDV IgA antibody in SICs.** SICs in the upper jejunum, lower jejunum and ileum were collected at 2, 7, 14, and 28 dpi from pigs inoculated with PEDV. The IgA ELISA was used to detect antibodies to PEDV in SICs collected from the upper jejunum, lower jejunum, and ileum. *p < 0.05 and **p < 0.01 (significant and highly significant differences between control and inoculated pigs euthanized at the indicated times, respectively) determined using Dunnett's test. ^‡^p < 0.05 (significant difference among tissues) determined using Tukey's test. Black bar: upper jejunum. Gray bar: lower jejunum. Open bar: ileum.

PEDV IgA antibodies were detected in the SICs of the upper jejunum, lower jejunum, and ileum at 14 and 28 dpi (Figure 3), with higher S/P ratios at 14 dpi than 28 dpi, which coincided with the decrease in viral shedding (Figure 1). The correlation coefficients between the PEDV IgA antibody S/P ratio in SICs of the upper jejunum, lower jejunum, and ileum and the number viral copies shed in fecal samples were r = − 0.932 (p = 0.068), r = − 0.986 (p = 0.014) and r = − 0.975 (p = 0.025), respectively. The PEDV IgA antibody S/P ratio in the SICs of the upper jejunum was significantly higher than that in SICs of the lower jejunum and ileum at 28 dpi, although no significant difference was observed among these sites at 14 dpi (Figure 3).

### Distribution of MNCs which actively produce PEDV IgA antibody

**Figure 4:**
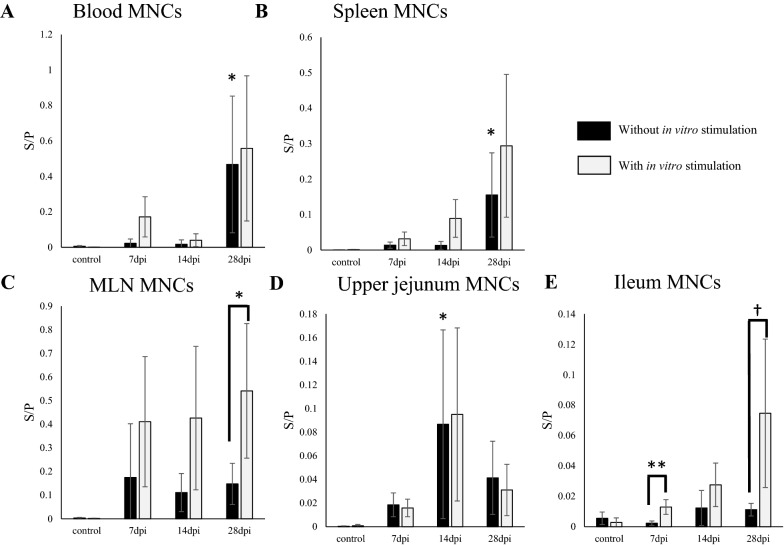
**S/P ratios of PEDV IgA antibody in culture supernatants of MNCs isolated from the blood, spleen, MLN, upper jejunum and ileum of PEDV-inoculated pigs, with or without in vitro stimulation.** MNCs were isolated from the blood (**A**), spleen (**B**), MLN (**C**), upper jejunum (**D**) and ileum (**E**) of PEDV-inoculated pigs. The MNCs were cultured for 4 days to evaluate the responses of specific ASCs. In the other wells, the MNCs were cultured with R848 for 4 days for stimulation of memory B cells. Cell supernatants were collected and PEDV-specific antibodies in the supernatants were detected by an IgA ELISA. *p < 0.05 vs. unstimulated group, by Dunnett's test. ^†^p < 0.1, *p < 0.05 and **p < 0.01 (suggestive, significant, and highly significant differences, respectively) for unstimulated vs. stimulated groups, by one-tailed Student's t-test. Black bar*:* without in vitro stimulation. Open bar: with in vitro stimulation.

To understand the systemic and intestinal distributions of PEDV IgG and IgA ASCs, we analyzed PEDV IgA and IgG antibody levels in the culture supernatants of MNCs isolated from systemic (blood and spleen) and mucosal (MLNs, upper jejunum and ileum) tissues without in vitro stimulation (Figure 4). PEDV IgA antibody was detected in the supernatants of MNCs isolated from the blood and spleen of PEDV-inoculated pigs euthanized at 7 dpi. At 28 dpi, PEDV IgA antibody levels from PEDV inoculated pigs were significantly higher than those from control pigs (Figure 4A, B). Similarly, PEDV IgA antibody was detected in the supernatants of MNCs isolated from the upper jejunum of PEDV-inoculated pigs euthanized as early as 7 dpi. PEDV IgA antibody levels were significantly enhanced in PEDV-inoculated pigs compared to control pigs at 14 dpi (Figure 4D), concurrently with the rise in the PEDV IgA antibody S/P ratio in SICs (Figure 3). A similar trend was observed for PEDV IgA antibody levels in the supernatants of MNCs from MLNs and the ileum, although no significant differences were observed between inoculated and control pigs (Figure 4C, E).

**Figure 5:**
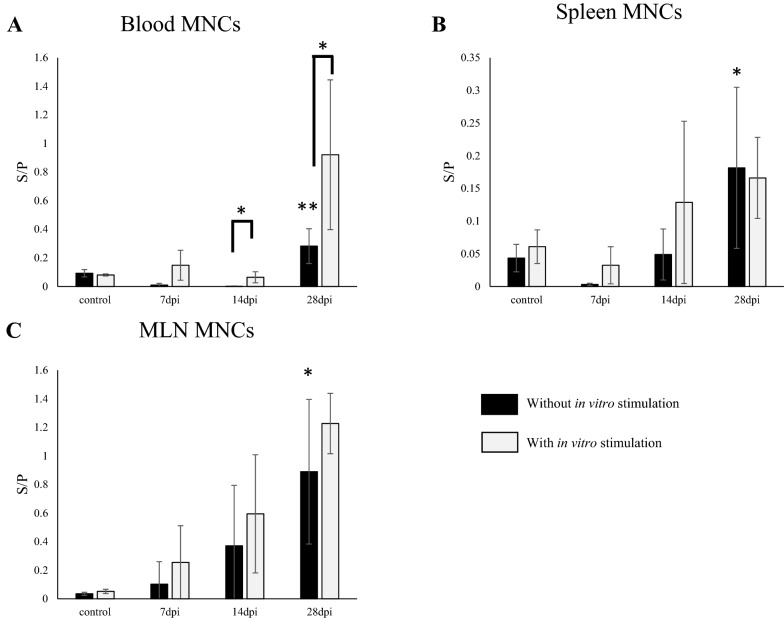
**S/P ratios of PEDV IgG antibody in culture supernatants of MNCs isolated from the blood, spleen and MLN of PEDV-inoculated pigs, with or without in vitro stimulation.** MNCs were isolated from the blood (**A**), spleen (**B**) and MLN (**C**) of PEDV inoculated pigs. MNCs were cultured for 4 days to evaluate the responses of specific ASCs. In the other wells, the MNCs were cultured with R848 for 4 days for the stimulation of memory B cells. Cell supernatants were collected, and PEDV-specific antibodies in the supernatants were detected by an IgG ELISA. *p < 0.05 and **p < 0.01 vs. the unstimulated group (significant and highly significant differences, respectively) by Dunnett's test. *p < 0.05 for unstimulated vs. stimulated groups, by one-tailed Student's t-test. Black bar: without in vitro stimulation. Open bar: with in vitro stimulation.

Although PEDV IgG antibody was detected as early as 14 dpi in serum (Figure 2A), secretion of PEDV IgG antibody from MNCs from the blood, spleen, and MLNs of PEDV-inoculated pigs was significantly increased at 28 dpi compared to that of control pigs (Figure 5). No PEDV IgG antibody secretion was observed from MNCs from the upper jejunum and ileum of PEDV-inoculated pigs throughout the experimental period (data not shown).

### Distribution of memory/resting B cells that can produce PEDV IgA or IgG antibodies upon in vitro stimulation

To investigate the distribution of memory/resting B cells, we stimulated isolated MNCs in vitro with R848, a TLR7/8 agonist that is known to stimulate polyclonal activation of memory B cells and induce antibody secretion [37]. In vitro stimulation had no effect on PEDV IgA and IgG antibody secretion by MNCs isolated from pigs in the control group (Figures 4, 5).

In contrast, in vitro stimulation of MNCs from PEDV-inoculated pigs significantly enhanced PEDV-specific IgA antibody secretion from MLNs at 28 dpi and the ileum at 7 dpi, and showed a trend towards enhancing secretion from the ileum at 28 dpi (p = 0.057), compared to no stimulation (Figure 4). These findings suggest that memory/resting B cells, which can produce PEDV IgA antibodies upon antigen stimulation, were present in the MLNs and ileum of PEDV-inoculated animals. In vitro stimulation did not significantly enhance PEDV IgA antibody secretion from MNCs isolated from the blood, spleen, or jejunum of PEDV-inoculated pigs compared to no stimulation (Figure 4A, B, D).

In vitro stimulation significantly increased the secretion of PEDV IgG antibodies from blood MNCs of PEDV-inoculated pigs euthanized at 14 and 28 dpi compared to no stimulation (Figure 5A), suggesting that memory/resting B cells which can produce PEDV IgG antibodies upon antigen stimulation were circulating in the blood at 28 dpi. No effect of in vitro stimulation was evident on PEDV IgG antibody secretion by MNCs isolated from the spleen (Figure 5B), MLNs (Figure 5C), or upper jejunum and ileum throughout the experiment (data not shown).

## Discussion

PED emerged or re-emerged globally in 2013–2015 and caused devastating economic losses to the pork industry. While, inactivated, live-attenuated, and other newly developed vaccines are available, many swine farmers choose to implement “controlled exposure”, in which animals are fed infectious PEDV contained in feces and/or intestinal homogenates from infected animals, to manage PED at their farms [38]. Understanding the mechanisms of antibody-mediated protection during PEDV infection is fundamental to the development of a better vaccine against PED. Here, we elucidated the systemic and intestinal IgA and IgG antibody responses and the distribution of IgA and IgG ASCs and memory B cells against PEDV in experimentally inoculated pigs over the course of 4 weeks.

Antibody levels in serum and/or fecal samples from infected animals are typically monitored to understand the humoral immune response, which plays a pivotal role in virus clearance and protection against subsequent re-infection. The present study showed that, at the same time that they were showing recovery from clinical signs and a decrease in virus shedding, more than half of the 16 virus-inoculated pigs had detectable PEDV IgA and IgG antibodies in their serum at 7 dpi. IgA and IgG antibody S/P ratios significantly increased after 14 dpi until 28 dpi in all inoculated pigs compared to control pigs (Figure 2). Similar results have been described by other research groups [39–41].

In contrast, PEDV IgA antibody was detected in fecal samples from less than half of the pigs until 14 dpi. While PEDV IgA antibody was detectable in all inoculated pigs at 28 dpi, levels were lower than those in serum and SICs (Figures 2B, 3). Similarly, lower PEDV IgA antibody levels in fecal samples were reported by Gerber et al*.* [28]. Although fecal sample collection has the advantage over serum sample collection of being noninvasive and easy to perform at any time by animal caretakers, the findings obtained in the present study and by other groups suggest that, compared to fecal samples, serum samples may provide more precise data regarding immune response.

We next investigated whether PEDV antibody responses in serum reflect those in the small intestine. In an experimental infection study, Tô et al*.* reported that the serum rotavirus IgA antibody level may serve as an indicator of the rotavirus IgA antibody level in the intestine of gnotobiotic pigs [25]. Similar to their findings, we observed fluctuations in PEDV IgA antibody levels in contents from the upper and lower jejunum and ileum that were similar to the fluctuations in serum: IgA antibody levels in SICs started to increase from 7–14 dpi and peaked at 28 dpi, when the highest IgA antibody level was observed in upper jejunal contents (Figure 3). The correlation coefficients between the S/P ratio of the upper jejunum, lower jejunum, and ileum IgA and serum IgA were r = 0.837 (p = 0.077), r = 0.934 (p = 0.020) and r = 0.910 (p = 0.032), respectively. Furthermore, the correlation coefficients between the S/P ratio of the upper jejunum, lower jejunum, and ileum IgA and serum IgG were r = 0.955 (p = 0.011), r = 0.997 (p < 0.001) and r = 0.991 (p = 0.001), respectively. These findings suggest that, at least during the 4-week period following PEDV inoculation, PEDV IgA and IgG antibody levels in serum may well reflect levels in the SICs.

Intestinal IgA antibodies are typically produced by plasma cells in the lamina propria and secreted into the gut lumen through cell-translocation via binding with polymeric immunoglobulin receptors [42]. To elucidate the distribution and antibody-producing levels of PEDV ASCs, we analyzed antibody levels in the culture supernatant of MNCs isolated from blood and tissues using ELISA. However, we did not determine the number of ASCs using an enzyme-linked immunospot (ELISPOT) assay, as was done in a previous PEDV study [27, 32, 35] and enteric virus studies [43, 44]. The decision to use ELISA but not ELISPOT was based on reports that antibody levels in culture supernatants of MNCs measured by ELISA or another method correlate well with the number of ASCs determined by ELISPOT in analyses of mucosal immune responses [45, 46]. In our present study, ASCs showing active production of PEDV IgA antibody without in vitro stimulation were identified in the upper jejunum but not in the ileum or MLNs of PEDV-inoculated pigs euthanized at 14 dpi, which coincided with the significant increase in PEDV IgA antibody levels in upper jejunum contents (Figures 3, 4). Considering together with previous findings that IgA-positive cell numbers were higher in the upper than lower small intestine in pigs [47], these findings may indicate that the upper jejunum is the major site of intestinal IgA production and secretion upon PEDV infection.

Memory B cells play important roles in the protection of individual animals from subsequent re-infection. We thus further analyzed the distribution of memory B cells in PEDV-inoculated pigs by examining the level of antibodies produced by MNCs isolated from blood and each tissue following in vitro stimulation. We stimulated isolated MNCs with the TLR7/8 agonist R848, which induces polyclonal memory B-cell expansion and antibody production without a specific antigen [37, 48–50]. Because B-cell responses can vary depending on the dose and virus proteins used for stimulation [32, 51], we hypothesized that in vitro stimulation with R848 may provide a clearer picture of memory B-cell responses to PEDV than stimulation with recombinant proteins or partially purified viruses.

Overall, compared with no stimulation, in vitro stimulation with R848 significantly enhanced PEDV IgA antibody secretion from MNCs isolated from MLNs at 28 dpi and the ileum significantly at 7 dpi, and showed a trend towards enhancing secretion from MNCs from the ileum at 28 dpi, the intestinal secondary lymphoid organ where naïve B-cell activation and differentiation take place (Figure 4). However, in vitro R848 stimulation did not enhance IgA antibody secretion from MNCs in the jejunum, where active production of the IgA antibody was observed without stimulation. These findings demonstrate the importance of MLNs and the ileum as inductive sites and the jejunum as an effective site of adaptive humoral immunity to PEDV infection.

Active production of PEDV IgA and IgG antibodies was also observed in culture supernatants of MNCs isolated from the blood and spleen of PEDV-inoculated pigs (Figure 4). Similarly, an earlier study also detected PEDV IgA and IgG ASCs in both systemic and intestinal tissues [32]. Possible explanations for this observation are that B cells that become activated and differentiated following activation by the virus are transported from the gut to the blood and other secondary lymphoid organs prior to antibody secretion [52], and/or B cells are activated in systemic secondary lymphoid tissues by PEDV antigens in systemic circulation [18, 53]. We observed lower IgA antibody levels in the culture supernatants of MNCs isolated from intestinal tissues than those from blood and spleen (Figure 3). This observation may be ascribable to the lower frequencies of B cells in intestinal tissues than in blood and spleen, as reported by Pasternak et al*.* [54]. However, further studies are required to clarify these phenomena.

In conclusion, we elucidated the kinetics of PEDV IgA and IgG antibody secretion and the distribution of ASCs in systemic and mucosal tissues. To our knowledge, this is the first report to investigate PEDV IgA antibody levels in various parts of the small intestine and to demonstrate the inductive and effective sites of the adaptive immune response in PEDV infection in young piglets in an experimental setting. Our findings will contribute to the understanding of immunity against enteric virus infection and the development of a more effective vaccine against PEDV.

## Data Availability

Not applicable.

## References

[1] Debouck P, Pensaert M (1980). Experimental infection of pigs with a new porcine enteric coronavirus, CV 777. Am J Vet Res.

[2] Li W, Li H, Liu Y, Pan Y, Deng F, Song Y, Tang X, He Q (2012). New variants of porcine epidemic diarrhea virus, China, 2011. Emerg Infect Dis.

[3] Pensaert MB, de Bouck P (1978). A new coronavirus-like particle associated with diarrhea in swine. Arch Virol.

[4] Wang D, Fang L, Xiao S (2016). Porcine epidemic diarrhea in China. Virus Res.

[5] Chen Q, Li G, Stasko J, Thomas JT, Stensland WR, Pillatzki AE, Gauger PC, Schwartz KJ, Madson D, Yoon K-J, Stevenson GW, Burrough ER, Harmon KM, Main RG, Zhang J (2014). Isolation and characterization of porcine epidemic diarrhea viruses associated with the 2013 disease outbreak among swine in the United States. J Clin Microbiol.

[6] Song D, Huang D, Peng Q, Huang T, Chen Y, Zhang T, Nie X, He H, Wang P, Liu Q, Tang Y (2015). Molecular characterization and phylogenetic analysis of porcine epidemic diarrhea viruses associated with outbreaks of severe diarrhea in piglets in Jiangxi, China 2013. PLoS One.

[7] Stevenson GW, Hoang H, Schwartz KJ, Burrough ER, Sun D, Madson D, Cooper VL, Pillatzki A, Gauger P, Schmitt BJ, Koster LG, Killian ML, Yoon KJ (2013). Emergence of porcine epidemic diarrhea virus in the United States: clinical signs, lesions, and viral genomic sequences. J Vet Diagn Invest.

[8] EFSA Panel on Animal Health and Welfare (2014). Scientific opinion on porcine epidemic diarrhoea and emerging pig deltacoronavirus. EFSA J.

[9] Masuda T, Murakami S, Takahashi O, Miyazaki A, Ohashi S, Yamasato H, Suzuki T (2015). New porcine epidemic diarrhoea virus variant with a large deletion in the spike gene identified in domestic pigs. Arch Virol.

[10] Sasaki Y, Alvarez J, Sekiguchi S, Sueyoshi M, Otake S, Perez A (2016). Epidemiological factors associated to spread of porcine epidemic diarrhea in Japan. Prev Vet Med.

[11] Kim S-H, Lee J-M, Jung J, Kim I-J, Hyun B-H, Kim H-I, Park C-K, Oem J-K, Kim Y-H, Lee M-H, Lee K-K (2015). Genetic characterization of porcine epidemic diarrhea virus in Korea from 1998 to 2013. Arch Virol.

[12] Park S, Kim S, Song D, Park B (2014). Novel porcine epidemic diarrhea virus variant with large genomic deletion, South Korea. Emerg Infect Dis.

[13] Dastjerdi A, Carr J, Ellis RJ, Steinbach F, Williamso S (2015). Porcine epidemic diarrhea virus among farmed pigs. Ukraine Emerg Infect Dis.

[14] Lee C (2015). Porcine epidemic diarrhea virus: an emerging and re-emerging epizootic swine virus. Virol J.

[15] Van Reeth K, Pensaert M (1994). Prevalence of infections with enzootic respiratory and enteric viruses in feeder pigs entering fattening herds. Vet Rec.

[16] Suzuki T, Shibahara T, Yamaguchi R, Nakade K, Yamamoto T, Miyazaki A, Ohashi S (2016). Pig epidemic diarrhea virus S gene variant with a large deletion non-lethal to colostrum-deprived newborn piglets. J Gen Virol.

[17] Saif LJ, Wang Q, Vlasova AN, Jung K, Xiao S, Zimmerman JJ, Karriker LA, Ramirez A, Schwartz KJ, Stevenson GW, Zhang J (2019). Coronaviruses. Diseases of swine.

[18] Madson DM, Arruda PHE, Magstadt DR, Burrough ER, Hoang H, Sun D, Bower LP, Bhandari M, Gauger PC, Stevenson GW, Wilberts BL, Wang C, Zhang J, Yoon KJ (2015). Characterization of porcine epidemic diarrhea virus isolate US/Iowa/18984/2013 infection in 1-day-old cesarean-derived colostrum-deprived piglets. Vet Pathol.

[19] Chen Q, Gauger PC, Stafne MR, Thomas JT, Madson DM, Huang H, Zheng Y, Li G, Zhang J (2016). Pathogenesis comparison between the United States porcine epidemic diarrhoea virus prototype and S-INDEL-variant strains in conventional neonatal piglets. J Gen Virol.

[20] Jung K, Annamalai T, Lu Z, Saif LJ (2015). Comparative pathogenesis of US porcine epidemic diarrhea virus (PEDV) strain PC21A in conventional 9-day-old nursing piglets vs. 26-day-old weaned pigs. Vet Microbiol.

[21] Lohse L, Krog JS, Strandbygaard B, Rasmussen TB, Kjær J, Belsham GJ, Bøtner A (2017). Experimental infection of young pigs with an early european strain of porcine epidemic diarrhoea virus and a recent US strain. Transbound Emerg Dis.

[22] Saif LJ (1996). Mucosal immunity: an overview and studies of enteric and respiratory coronavirus infections in a swine model of enteric disease. Vet Immunol Immunopathol.

[23] Pierre JF, Busch RA, Kudsk KA (2016). The gastrointestinal immune system: implications for the surgical patient. Curr Probl Surg.

[24] Saif LJ, van Cott JL, Brim TA (1994). Immunity to transmissible gastroenteritis virus and porcine respiratory coronavirus infections in swine. Vet Immunol Immunopathol.

[25] Tô TL, Ward LA, Yuan L, Saif LJ (1998). Serum and intestinal isotype antibody responses and correlates of protective immunity to human rotavirus in a gnotobiotic pig model of disease. J Gen Virol.

[26] Ward LA, Yuan L, Rosen BI, Tô TL, Saif LJ (1996). Development of mucosal and systemic lymphoproliferative responses and protective immunity to human group A rotaviruses in a gnotobiotic pig model. Clin Diagn Lab Immunol.

[27] Langel SN, Paim FC, Alhamo MA, Lager KM, Vlasova AN, Saif LJ (2019). Oral vitamin A supplementation of porcine epidemic diarrhea virus infected gilts enhances IgA and lactogenic immune protection of nursing piglets. Vet Res.

[28] Gerber PF, Opriessnig T (2015). Detection of immunoglobulin (Ig) A antibodies against porcine epidemic diarrhea virus (PEDV) in fecal and serum samples. MethodsX.

[29] Cebra JJ, Fuhrman JA, Griffin P, Rose FV, Schweitzer PA, Zimmerman D (1984). Changes in specific B cells and the dissemination of the primed state in vivo following antigenic stimulation by different mucosal routes. Ann Allergy.

[30] Brandtzaeg P (1992). Humoral immune response patterns of human mucosae: induction and relation to bacterial respiratory tract infections. J Infect Dis.

[31] Mestecky J (1987). The common mucosal immune system and current strategies for induction of immune responses in external secretions. J Clin Immunol.

[32] de Arriba ML, Carvajal A, Pozo J, Rubio P (2002). Isotype-specific antibody-secreting cells in systemic and mucosal associated lymphoid tissues and antibody responses in serum of conventional pigs inoculated with PEDV. Vet Immunol Immunopathol.

[33] Suzuki T, Murakami S, Takahashi O, Kodera A, Masuda T, Itoh S, Miyazaki A, Ohashi S, Tsutsui T (2015). Molecular characterization of pig epidemic diarrhoea viruses isolated in Japan from 2013 to 2014. Infect Genet Evol.

[34] Stadler J, Zoels S, Fux R, Hanke D, Pohlmann A, Blome A, Weissenböck H, Weissenbacher-Lang C, Ritzmann M, Ladinig A (2015). Emergence of porcine epidemic diarrhea virus in southern Germany. BMC Vet Res.

[35] de Arriba ML, Carvajal A, Pozo J, Rubio P (2002). Mucosal and systemic isotype-specific antibody responses and protection in conventional pigs exposed to virulent or attenuated porcine epidemic diarrhoea virus. Vet Immunol Immunopathol.

[36] Ouyang K, Shyu D-L, Dhakal S, Hiremath J, Binjawadagi B, Lakshmanappa YS, Guo R, Ransburgh R, Bondra KM, Gauger P, Zhang J, Specht T, Gilbertie A, Minton W, Fang Y, Renukaradhya GJ (2015). Evaluation of humoral immune status in porcine epidemic diarrhea virus (PEDV) infected sows under field conditions. Vet Res.

[37] Jahnmatz M, Kesa G, Netterlid E, Buisman A-M, Thorstensson R, Ahlborg N (2013). Optimization of a human IgG B-cell ELISpot assay for the analysis of vaccine-induced B-cell responses. J Immunol Methods.

[38] Niederwerder MC, Hesse RA (2018). Swine enteric coronavirus disease: a review of 4 years with porcine epidemic diarrhoea virus and porcine deltacoronavirus in the United States and Canada. Transbound Emerg Dis.

[39] Gimenez-Lirola LG, Zhang J, Carrillo-Avila JA, Chen Q, Magtoto R, Poonsuk K, Baum DH, Piñeyro P, Zimmerman J (2017). Reactivity of porcine epidemic diarrhea virus structural proteins to antibodies against porcine enteric coronaviruses: diagnostic implications. J Clin Microbiol.

[40] Krishna VD, Kim Y, Yang M, Vannucci F, Molitor T, Torremorell M, Cheeran MC-J (2020). Immune responses to porcine epidemic diarrhea virus (PEDV) in swine and protection against subsequent infection. PLoS One.

[41] Lin C-M, Ghimire S, Hou Y, Boley P, Langel PN, Vlasova AN, Saif LJ, Wang Q (2019). Pathogenicity and immunogenicity of attenuated porcine epidemic diarrhea virus PC22A strain in conventional weaned pigs. BMC Vet Res.

[42] Turula H, Wobus CE (2018). The role of the polymeric immunoglobulin receptor and secretory immunoglobulins during mucosal infection and immunity. Viruses.

[43] Yuan L, Ward LA, Rosen BI, To TL, Saif LJ (1996). Systematic and intestinal antibody-secreting cell responses and correlates of protective immunity to human rotavirus in a gnotobiotic pig model of disease. J Virol.

[44] Carter MJ, Mitchell RM, Sauteur PMM, Kelly DF, Trück J (2017). The antibody-secreting cell response to infection: kinetics and clinical applications. Front Immunol.

[45] Carpenter CM, Hall ER, Randall R, McKenzie R, Cassels F, Diaz N, Thomas N, Bedford P, Darsley M, Gewert C, Howard C, Sack RB, Sack DA, Chang HS, Gomes G, Bourgeois AL (2006). Comparison of the antibody in lymphocyte supernatant (ALS) and ELISPOT assays for detection of mucosal immune responses to antigens of enterotoxigenic *Escherichia coli* in challenged and vaccinated volunteers. Vaccine.

[46] Feller AJ, McKenzie R, Taylor DN, Woods CC, Grahek SL, Islam D, Venkatesan MM, Hale TL, Bourgeois AL (2011). Comparative evaluation of the antibody in lymphocyte supernatant (ALS) and enzyme-linked immunospot (ELISPOT) assays for measuring mucosal immune responses to Shigella antigens. Vaccine.

[47] Allen WD, Porter P (1973). The relative distribution of IgM and IgA cells in intestinal mucosa and lymphoid tissues of the young unweaned pig and their significance in ontogenesis of secretory immunity. Immunology.

[48] Christiansen D, Earnest-Silveira L, Grubor-Bauk B, Wijesundara DK, Boo I, Ramsland PA, Vincan E, Drummer HE, Gowans EJ, Torresi J (2019). Pre-clinical evaluation of a quadrivalent HCV VLP vaccine in pigs following microneedle delivery. Sci Rep.

[49] Pinna D, Corti D, Jarrossay D, Sallusto F, Lanzavecchia A (2009). Clonal dissection of the human memory B-cell repertoire following infection and vaccination. Eur J Immunol.

[50] Auladell M, Nguyen TH, Garcillán B, Mackay F, Kedzierska K, Fox A (2019). Distinguishing naive- from memory-derived human B cells during acute responses. Clin Transl Immunol.

[51] Yuan L, Geyer A, Saif LJ (2001). Short-term immunoglobulin A B-cell memory resides in intestinal lymphoid tissues but not in bone marrow of gnotobiotic pigs inoculated with Wa human rotavirus. Immunology.

[52] Langel SN, Wang Q, Vlasova AN, Saif LJ (2020). Host factors affecting generation of immunity against porcine epidemic diarrhea virus in pregnant and lactating swine and passive protection of neonates. Pathogens.

[53] Jung K, Wang Q, Scheuer KA, Lu Z, Zhang Y, Saif LJ (2014). Pathology of US porcine epidemic diarrhea virus strain PC21A in gnotobiotic pigs. Emerg Infect Dis.

[54] Pasternak JA, Ng SH, Kaser T, Meurens F, Wilson HL (2014). Grouping pig-specific responses to mitogen with similar responder animals may facilitate the interpretation of results obtained in an out-bred animal model. J Vacc Vaccinol.

